# The *BIM* deletion polymorphism: A paradigm of a permissive interaction between germline and acquired TKI resistance factors in chronic myeloid leukemia

**DOI:** 10.18632/oncotarget.5436

**Published:** 2015-10-28

**Authors:** Tun Kiat Ko, Hui San Chin, Charles T.H. Chuah, John W.J. Huang, King-Pan Ng, Seong Lin Khaw, David C.S. Huang, S. Tiong Ong

**Affiliations:** ^1^ Cancer and Stem Cell Biology Program, Duke-NUS Graduate Medical School, Singapore; ^2^ The Walter and Eliza Hall Institute of Medical Research, Parkville, VIC, Australia; ^3^ Department of Medical Biology, University of Melbourne, Parkville, VIC, Australia; ^4^ Department of Haematology, Singapore General Hospital, Singapore; ^5^ Royal Children's Hospital, Parkville, VIC, Australia; ^6^ Department of Medical Oncology, National Cancer Centre, Singapore; ^7^ Department of Medicine, Duke University Medical Center, Durham, NC, USA; ^8^ Present address: Singapore Institute for Clinical Sciences (SICS), Brenner Centre for Molecular Medicine, Singapore; ^9^ Present address: Cancer Science Institute of Singapore, National University of Singapore, Singapore

**Keywords:** BIM deletion polymorphism, CML, TKI resistance, BH3 mimetic, BCR-ABL1

## Abstract

Both germline polymorphisms and tumor-specific genetic alterations can determine the response of a cancer to a given therapy. We previously reported a germline deletion polymorphism in the *BIM* gene that was sufficient to mediate intrinsic resistance to tyrosine kinase inhibitors (TKI) in chronic myeloid leukemia (CML), as well as other cancers [1]. The deletion polymorphism favored the generation of *BIM* splice forms lacking the pro-apoptotic BH3 domain, conferring a relative resistance to the TKI imatinib (IM). However, CML patients with the BIM deletion polymorphism developed both partial and complete IM resistance. To understand the mechanisms underlying the latter, we grew CML cells either with or without the *BIM* deletion polymorphism in increasing IM concentrations. Under these conditions, the *BIM* deletion polymorphism enhanced the emergence of populations with complete IM resistance, mimicking the situation in patients. Importantly, the combined use of TKIs with the BH3 mimetic ABT-737 overcame the *BCR-ABL1*-dependent and -independent resistance mechanisms found in these cells. Our results illustrate the interplay between germline and acquired genetic factors in confering TKI resistance, and suggest a therapeutic strategy for patients with complete TKI resistance associated with the *BIM* deletion polymorphism.

## INTRODUCTION

The *BCR-ABL1* gene fusion, a product of a chromosomal translocation involving chromosomes 9 and 22 [2, 3], encodes for a constitutively active tyrosine kinase that drives the pathogenesis of chronic myeloid leukemia (CML) [4–9]. Germline polymorphisms and tumor-specific genetic mutations independently contribute to the behavior of human cancers, including the response to therapy. However, few specific models allow for the detailed study of how inherited and acquired genetic factors might interact to cause clinical drug resistance, nor how their interaction can be prevented or overcome.

We recently reported a germline deletion polymorphism in the *BIM* gene that was sufficient to mediate intrinsic resistance to targeted therapies in cancer, including the examples of imatinib (IM) in CML and EGFR inhibitors in EGFR-mutated non-small cell lung cancer (EGFR-NSCLC) [1]. *BIM,* also known as *BCL2L11*, encodes for a BH3-only protein and it is a member of the *BCL2* protein family. The BH3-only proteins activate apoptosis by either opposing the pro-survival members of the *BCL2* family (e.g. *BCL2, BCL-XL*, and *MCL1*), or by binding to the pro-apoptotic *BCL-2* family members (e.g. *BAX* and *BAK1*) and directly activating their pro-apoptotic functions [10]. Importantly, CML cells maintain a survival advantage by suppressing *BIM* transcription and by targeting *BIM* for proteasomal degradation through *MAPK1*-dependent phosphorylation [11–13]. Additionally, *BIM* up-regulation is required for TKIs to induce apoptosis, and suppression of *BIM* expression is sufficient to confer *in vitro* TKI resistance [11–13].

**Figure 1:**
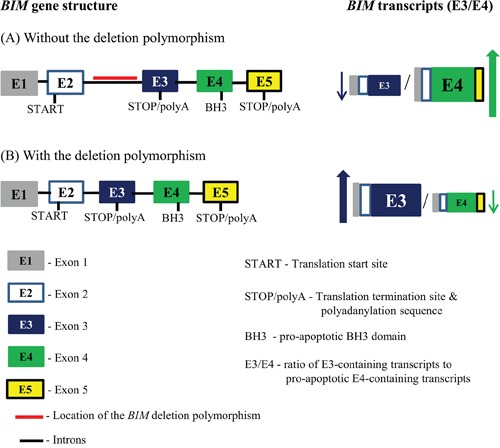
The location of the *BIM* deletion polymorphism within the *BIM* gene and its effect on splicing of *BIM* transcripts The *BIM* gene is illustrated showing the distribution of both the introns and the major exons. The deleted region, highlighted with a red line, constitutes the *BIM* deletion polymorphism which is located in the intron found between exons 2 and 3. Exon 4 encodes for the BH3 domain that is required for BIM apoptotic function, whereas exon 3 lacks this domain. Since exon 3 and exon 4 undergo mutually exclusive splicing, exon-3-containing transcripts will not contain a BH3 domain. The *BIM* gene without **A.** and with **B.** the *BIM* deletion polymorphism as well as the effect of the *BIM* deletion polymorphism has on the ratio of E3-containing transcripts to pro-apoptosis E4-containing transcripts (E3/E4) are illustrated. The *BIM* gene without the deletion polymorphism (A) results in a lower E3/E4 ratio when compared to one with the deletion polymorphism (B), where the shortened intron between exons 2 and 3 results in increased splicing preference for exon3 over exon 4, and hence results in a higher E3/E4 ratio. The *BIM* gene is not drawn to scale.

The *BIM* deletion polymorphism consists of a 2,903-bp deleted region that is found in the intron found between exons 2 and 3 of the *BIM* gene (Figure 1A) [1]. Mechanistically, the *BIM* deletion polymorphism leads to the preferential generation of *BIM* splice forms that lack the pro-apoptotic BH3 domain, and are thus incapable of activating apoptosis in response to targeted therapy (Figure 1) [1]. Accordingly, TKI-sensitive CML cell lines genetically engineered to contain the deletion expressed less pro-apoptotic BH3-containing *BIM* isoforms upon exposure to imatinib, resulting in an impaired apoptotic response to TKIs, and a relative TKI resistance [1].

Clinically, and as predicted from our cell line data, we found that CML patients with the *BIM* deletion polymorphism had inferior first-line responses to standard dose IM compared to patients without the deletion [1]. Furthermore, among the 26 patients with the *BIM* deletion who experienced inferior responses, only four (15%) were found to have *ABL* kinase domain mutations associated with TKI-resistance [1]. The presence of kinase domain mutations among patients with the *BIM* deletion polymorphism who developed clinical resistance, as well as the cross-resistance to second-generation TKIs experienced by half the patients with the polymorphism [1], suggested that the *BIM* deletion polymorphism might be cooperating with other resistance-conferring mechanisms acquired during TKI exposure to produce the observed TKI resistance.

To better understand the relationship between the *BIM* deletion polymorphism and acquired TKI resistance mechanisms, we used a cell line-based approach to first induce high levels of TKI resistance [14–19], and then used these cells to uncover the underlying TKI-resistance mechanisms that cooperate with the *BIM* deletion polymorphism to confer TKI resistance. Here, we report that the *BIM* deletion polymorphism is permissive for the acquisition of somatic TKI-resistance conferring events that are both dependent and independent of *BCR-ABL1*, and identify a therapeutic strategy to overcome *BIM* deletion polymorphism-associated TKI-resistance.

## RESULTS

### The *BIM* deletion polymorphism significantly enhances the viability of K562 clones in the presence of high-dose imatinib

**Figure 2:**
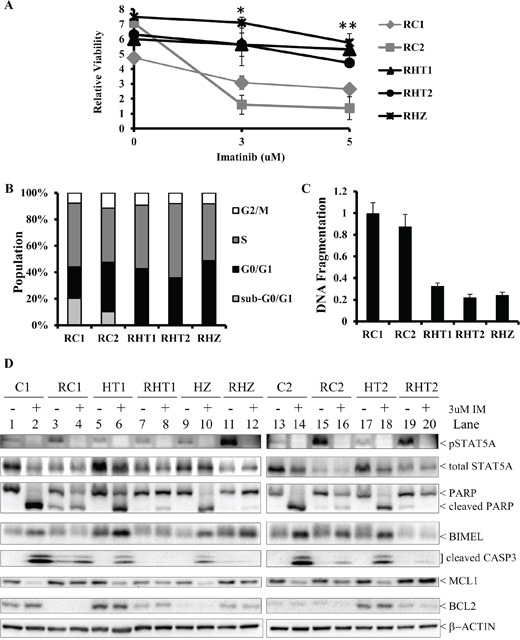
Imatinib-resistant K562 clones with the *BIM* deletion polymorphism are more viable in high imatinib concentration than their non-*BIM* deletion polymorphism-containing counterparts **A.** The relative viability, as measured by MTS assay, of the different imatinib-resistant cells following 6 days of exposure to imatinib at 0, 3, and 5 uM concentration. The relative viability was calculated as a ratio of the viability at day 6 to that at day 0. Results are given as mean +/− s.e.m (*n* = 3). The *P* values were based on Student's *t* test. The *P* value for each of the imatinib concentration was calculated by comparing the relative viability of RHT1, RHT2 and RHZ to those of RC1 and RC2. *P = 0.0204, **P = 0.0209. **B.** The cell cycle profile for the different imatinib-resistant clones by propidium iodide (PI) staining. Results are given as mean (*n* = 3). **C.** Imatinib-resistant K562 clones with the *BIM* deletion polymorphism had less apoptotic activity than their non-*BIM* deletion polymorphism-containing counterparts. The ELISA-based DNA fragmentation assay was used to measure cellular apoptosis, as previously described [1]. The DNA fragmentation value is a ratio of the reading for a given sample to that of RC1. Results are given as mean +/− s.e.m (*n* = 3). **D.** Immunoblots of cell lysates from parental clones (C1, C2, HT1, HT2 & HZ) and their corresponding imatinib-resistant counterparts (RC1, RC2, RHT1, RHT2 & RHZ) following culture with either DMSO (−) or 3 uM IM (+) for 48 hours.

Previously, we reported that CML patients with the *BIM* (Table 1) deletion polymorphism were at increased risk of experiencing inferior imatinib responses compared to those without [1]. Furthermore, among patients with inferior imatinib responses, a proportion developed resistance to the more potent second-generation TKIs, and progressed to blast crisis [1]. This clinical observation was unexpected given that the *BIM* deletion polymorphism confers a relative and not absolute resistance to TKIs [1]. To explain this observation, we hypothesized that the germline *BIM* deletion polymorphism enhances the acquisition of somatic TKI-resistance mutations, which then together, cooperate to produce higher levels of TKI resistance, including cross-resistance to the more potent second generation TKIs. To test this hypothesis, we cultured genome-edited K562 clones, either with or without the *BIM* deletion polymorphism, in increasing doses of IM over a 4-month period (Table 1). By the end of 4 months, we found that cells harboring the *BIM* deletion polymorphism were more viable at ranges of imatinib (3 to 5 uM) corresponding to the maximal plasma imatinib concentrations tolerated by patients [20, 21]. Thus, as depicted in Figure 2A, at 3 and 5 uM imatinib, all three IM-resistant clones with the *BIM* deletion polymorphism (RHT1, RHT2 and RHZ) were three to five times more viable than those (RC1 and RC2) without the polymorphism (Figure 2A). We then used these IM-resistant K562 clones to study the molecular relationship between imatinib resistance and the *BIM* deletion polymorphism.

**Table 1:** A guide to the different imatinib-sensitive parental K562 clones and their corresponding imatinib-resistant (IMR) clones as well as their *BIM* deletion polymorphism status ‘HET’ and ‘HOM’ indicate K562 clones that are heterozygous and homozygous for BIM deletion polymorphism respectively. Note that regular K562 does not carry the BIM deletion polymorphism. K562 clones indicated below that do carry the BIM deletion polymorphism were generated by using zinc finger nuclease (ZFN)-mediated genome editing (see main text for details)

*BIM* deletion polymorphism status		Parental Clones	Corresponding IMR clones
without		C1	RC1
		C2	RC2
with	HET	HT1	RHT1
		HT2	RHT2
	HOM	HZ	RHZ

Since the MTS viability assay reflects changes in both cell survival as well as cell proliferation, we next measured these two parameters directly. We used flow cytometry and PI staining to assess the cell cycle profile of each IM-resistant clones. Generally, there was no significant difference in the cell cycle profiles for all the imatinib-resistant clones that were cultured long-term in 3 uM imatinib (Figure 2B). In conclusion, there was no significant change in cell proliferation among the imatinib-resistant clones. To assay for apoptosis, we used an ELISA-based DNA fragmentation assay that detects monomeric and oligomeric nucleosomes that are generated by apoptosis-activated nucleases [1]. Compared to their counterparts without the *BIM* deletion polymorphism, imatinib-resistant clones with the polymorphism exhibited, on average, a three-fold reduction in cell death, as measured by the amount of apoptosis-induced fragmented DNA when all the clones were cultured long-term in 3 uM imatinib (Figure 2C). Moreover, consistent with the DNA fragmentation assay (Figure 2C), we found that there were more apoptotic cells, as indicated by the presence of a significant sub-G1 population, in imatinib-resistant clones without the polymorphism (Figure 2B).

To confirm that the *BIM* deletion polymorphism-containing imatinib-resistant clones had impaired apoptotic response to imatinib, we performed immunoblotting on all imatinib-resistant clones and, as controls, their corresponding imatinib-sensitive parental clones (Figure 2D). In the presence of 3 uM imatinib, apoptosis was clearly induced in all the imatinib-sensitive parental clones (C1, C2, HT1, HT2, HZ) as evidenced by cleaved PARP, cleaved CASPASE 3, and elevated BIMEL expression (Figure 2D; compare lanes 1, 5, 9, 13, and 17 to lanes 2, 6, 10, 14, and 18 respectively). In contrast to their imatinib-sensitive parental clones, the corresponding imatinib-resistant clones were more resistant to imatinib-induced apoptosis, as evidenced by the presence of significantly less cleaved PARP and cleaved CASPASE 3 (Figure 2D; compare lanes 2, 6, 10, 14 and 18 to lanes 4, 8, 12, 16 and 20 respectively). Among the imatinib-resistant clones, we also observed that clones with the polymorphism were more resistant to imatinib-induced apoptosis when compared to those without the polymorphism (Figure 2D; compare lanes 4 and 16 to lanes 8, 12 and 20). In conclusion, we find that *BIM* deletion polymorphism facilitates the emergence of imatinib-resistant clones, inlcuding clones which exhibit complete resistance to the highest concentrations of imatinib that can be tolerated by patients.

### The *BIM* deletion polymorphism cooperates with somatic TKI resistance mechanisms

To understand the mechanism behind their impaired response to imatinib-induced apoptosis, we determined if the presence of the *BIM* deletion polymorphism is permitting and/or cooperating with known mechanisms of TKI resistance which include both BCR-ABL1-dependent and -independent mechanisms. For BCR-ABL1-dependent TKI resistance, we investigated whether *BCR-ABL1* somatic mutations and/or BCR-ABL1 overexpression were involved. As for BCR-ABL1-independent TKI resistance, we determined whether ERK and/or LYN activation were present, as previously described [15, 16, 22–24].

**Figure 3:**
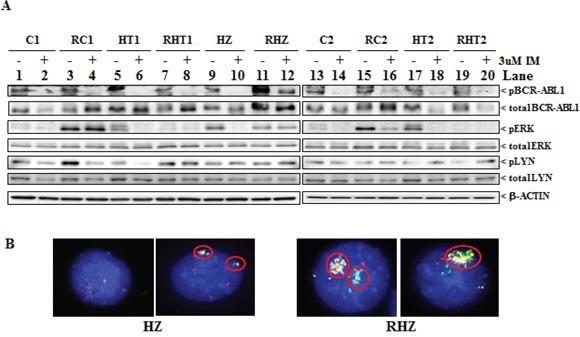
Characteristics of imatinib-resistant clones **A.** Immunoblots of cell lysates from parental clones (C1, C2, HT1, HT2 & HZ) and their corresponding imatinib-resistant counterparts (RC1, RC2, RHT1, RHT2 & RHZ) following culture with either DMSO (−) or 3 uM IM (+) for 48 hours. Antibodies used were phosphorylated BCR-ABL1 (pBCR-ABL1), total BCR-ABL1, phosphorylated ERK (pERK), phosphorylated LYN (pLYN), total ERK and total LYN. **B.** Representative images of fluorescence *in situ* hybridisation (FISH) performed on parental HZ and its corresponding imatinib-resistant counterpart, RHZ, demonstrating amplification of the BCR-ABL1 fusion gene locus (circled) in RHZ when compared to parental HZ.

Previous reports showed that somatic mutations within the kinase domain of BCR-ABL1 can result in IM resistance [25–28]. Therefore, we examined the effect of imatinib on the level of phosphorylation of tyrosine-245 in the *ABL1* kinase domain, an indicator of activated ABL1 kinase [29]. In parental clones, the addition of IM resulted in the loss of phospho-BCR-ABL1 signal (Figure 3A, panel ‘pBCR-ABL1’ compare lanes 1, 5, 9, 13 and 17 to lanes 2, 6, 10, 14 and 18 respectively). Except for RHZ, long-term culture in imatinib also resulted in the loss of phospho-BCR-ABL1 signal in imatinib-resistant cells (Figure 3A, panel ‘pBCR-ABL1’ compare lane 12 to lanes 4, 8, 16 and 20). Since significant amounts of BCR-ABL1 in RHZ remained phosphorylated despite the presence of imatinib, we decided to sequence the *BCR-ABL1* kinase domain of all the imatinib-resistant clones. As expected, only RHZ carried a mutation within the *BCR-ABL1* kinase domain (Table 2). The mutation occurred at glycine-250 (G250), which was mutated to glutamate (E). Mutation at this position (G250E) is known to confer resistance to imatinib with a reported IC_50_ of at least 7 uM when the mutant BCR-ABL1 was expressed in Ba/F3 cells [26, 27]. We also noted that, compared to the other imatinib-resistant cells, the level of BCR-ABL1 protein expression in RHZ was significantly higher (Figure 3A, panel ‘total BCR-ABL1’; lanes 11 & 12). It has been reported that BCR-ABL1 gene overexpression is another known mechanism that contributes to imatinib resistance and normally, BCR-ABL1 overexpression correlates with amplification of the *BCR-ABL1* gene [25]. Accordingly, we performed fluorescence *in situ* hybridization (FISH) on both parental HZ and imatinib-resistant RHZ (Figure 3B), and found that there were more RHZ cell that showed significant amplification of the *BCR-ABL1* gene when compared to parental HZ cells (Supplementary Table S3).

**Table 2:** A summary of the different BCR-ABL1-dependent and -independent mechanisms of TKI resistance that were acquired by the different imatinib-resistant (IMR) clones The BIM deletion polymorphism status of the IMR clones is indicated below. ‘HET’ and ‘HOM’ indicate IMR clones that are heterozygous and homozygous for the BIM deletion polymorphism respectively. Note that “−“ denotes absent; “+”, “++” and “+++” denote increasing strength of activity or presence

	IMR cells (*BIM* deletion polymorphism status)		
TKI resistance mechanism	without	with	
		HET	HOM
Somatic mutation in BCR-ABL1	−	−	G250E
BCR-ABL1 gene amplification	−	−	+++
LYN activation	+	+++	+
ERK activation	+++	−	++

Activation of the ERK pathway is another known mechanism that contributes to imatinib resistance [15, 23]. Here, we found that total levels of ERK remain unchanged between resistant lines and the parental counterparts (Figure 3A, panel ‘total ERK’). However, we observed that the increased level of phosphorylated ERK (pERK), which is a readout for activated ERK, could be observed in three (RC1, RC2 and RHZ) out of the five resistant cell lines (Figure 3A, panel ‘pERK’). In RC1, the increased ERK phosphorylation could not be inhibited by IM despite effective IM-mediated BCR-ABL1 inhibition, whereas it could be inhibited in RC2 (Figure 3A, panel ‘pERK’; compare lanes 3 and 15 to lanes 4 and 16 respectively). Among the *BIM* deletion polymorphism-containing imatinib-resistant cells, RHZ exhibited persistent ERK activation (Figure 3A, panel ‘pERK’; lanes 11 and 12), but unlike RC1, this was most likely due to the inability of imatinib to completely inhibit the G250E mutation present in RHZ (Figure 3A, panel ‘pBCR-ABL1’; compare lanes 11 and 12 to lanes 9 and 10 respectively).

Overexpression and activation of the SRC family of kinases (SFK), especially LYN, is another known mechanism mediating imatinib resistance [16, 22, 24]. Phosphorylated LYN (pLYN), an indicator of activated LYN, was observed in all the imatinib-resistant clones albeit at different levels of expression (Figure 3A, panel ‘pLYN’). Importantly, among the two resistant clones with increased pLYN compared to their parental counterparts (RC1 and RHT1), IM treatment was not able to decrease phosphorylated LYN in RHT1 cells but was able to reduce phosphorylated BCR-ABL1, suggesting that LYN activation was BCR-ABL1-independent (Figure 3A, panel ‘pLYN’; compare lane 7 to lane 8). Taken together, our data show that the *BIM* deletion polymorphism facilitates the development of imatinib resistance, which is in turn underwritten by mechanisms that are both BCR-ABL1-dependent and -independent (Table 2).

### The use of second-generation TKIs and the BH3-mimetic ABT-737 overcomes TKI resistance in *BIM* deletion polymorphism-containing imatinib-resistant cells

Since we found that the *BIM* deletion polymorphism cooperates with both BCR-ABL1-dependent and -independent mechanisms of TKI resistance, we next set out to determine whether second-generation TKIs, such as dasatinib and nilotinib, could be used as a therapeutic strategy to overcome these TKI resistance mechanisms. Dasatinib and nilotinib can overcome some of the known TKI resistance mechanisms such as somatic mutations in the *BCR-ABL1* kinase domain and activated LYN [30, 31]. Additionally, we had previously shown that combined treatment with both the BH3-mimetic ABT-737 and imatinib resensitized parental K562 with the polymorphism to imatinib-induced apoptosis [1]. Thus, we wished to determine whether treatment with second-generation TKIs and/or ABT-737 could overcome TKI resistance in *BIM* deletion polymorphism-containing imatinib-resistant clones.

**Figure 4:**
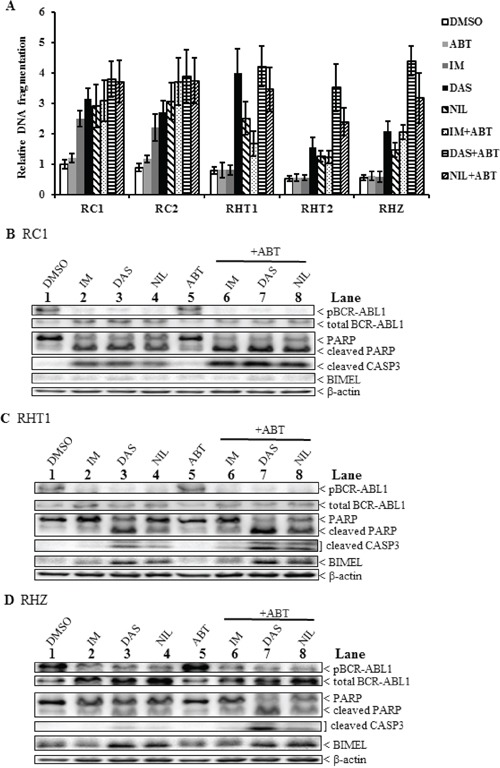
A combination of a second-generation TKI and the BH3 mimetic ABT-737 induced maximal apoptosis in imatinib-resistant *BIM* deletion polymorphism-containing clones **A.** ELISA-based DNA fragmentation assay for the different imatinib-resistant clones incubated with or without TKIs, alone or in combination with ABT-737 (ABT, 2.5 uM) for 48 hours. Equipotent amounts of each TKI were used: 3 uM imatinib (IM), 30 nM dasatinib (DAS), and 300 nM nilotinib (NIL). The DNA fragmentation value is the ratio of the reading for a given sample to that of RHT2 treated with IM. Results are given as mean +/− s.e.m (*n* = 3). **B–D.** Immunoblots of selected imatinib-resistant clones (B, RC1; C, RHT1; D, RHZ) incubated with different TKIs, alone or in combination with ABT-737 (ABT, 2.5 uM), for 48 hours before harvesting. Equipotent amounts of each TKI were used: 3 uM imatinib (IM), 30 nM dasatinib (DAS), and 300 nM nilotinib (NIL).

We used an ELISA-based DNA fragmentation assay as a read-out for apoptosis in imatinib-resistant clones that were treated with either or both second-generation TKIs and ABT-737 (Figure 4A). For imatinib-resistant clones without the polymorphism (RC1 and RC2), treatments with equipotent amount of second-generation TKIs resulted in a 30% increase, on average, in apoptosis when compared to imatinib alone (Figure 4A and Supplementary Table S1). As for *BIM* deletion polymorphism-containing imatinib-resistant clones (RHT1, RHT2 and RHZ), treatments with equipotent amounts of second-generation TKIs induced a dramatically 220% increase, on average, in apoptosis when compared to imatinib alone (Figure 4A and Supplementary Table S1). For imatinib-resistant clones without the polymorphism, treatments with both imatinib and ABT-737 resulted in a 50% increase, on average, in apoptosis when compared to imatinib alone (Figure 4A and Supplementary Table S1). As for *BIM* deletion polymorphism-containing imatinib-resistant clones, treatments with both imatinib and ABT-737 significantly induced a 160% increase, on average, in apoptosis when compared to imatinib alone (Figure 4A and Supplementary Table S1). For imatinib-resistant clones without the polymorphism, treatments with both second-generation TKIs and ABT-737 resulted in a 60% increase, on average, in apoptosis when compared to imatinib alone (Figure 4A and Supplementary Table S1). As for *BIM* deletion polymorphism-containing imatinib-resistant clones, treatments with both second-generation TKIs and ABT-737 dramatically induced a 450% increase, on average, in apoptosis when compared to imatinib alone (Figure 4A and Supplementary Table S1).

We also performed immunoblotting on imatinib-resistant clones that were treated with either or both second-generation TKIs and ABT-737. The results reflected those generated from the ELISA-based fragmentation assays mentioned earlier. For imatinib-resistant clones without the polymorphism, maximal apoptosis was induced when cells were treated with a combination of TKIs and ABT-737 as evident by the presence of significantly higher amount of cleaved CASPASE3 and cleaved PARP when compared to those treated with either TKIs or ABT-737 alone (Figure 4B; compare lanes 6–8 to lanes 2–5). As for *BIM* deletion polymorphism-containing imatinib-resistant clones, maximal apoptosis was induced when cells were treated with a combination of second-generation TKIs, especially dasatinib, and ABT-737 as evident by the presence of the highest level of both cleaved CASPASE3 and cleaved PARP when compared to the others (Figures 4C and 4D, compare lane 7 to lanes 3, 4 and 8). For all three imatinib-resistant clones, we also observed that BIMEL level was further induced in samples treated with second-generation TKIs, especially dasatinib (Figure 4B, lanes 3 and 7; Figure 4C & 4D, lanes 3, 4, 7 and 8). In conclusion, the use of second-generation TKIs and BH3 mimetic ABT-737 had a significant effect on overcoming TKI resistance in imatinib-resistant clones, especially in those that harbored the polymorphism, where the effects were dramatic (Figure 4A and Supplementary Table S1). Furthermore, we observed that the increased apoptosis was correlated with increased expression of BIMEL, an important regulator of apoptosis in CML.

### Combination of the BH3-mimetic ABT-737 and a TKI induced maximal apoptosis in imatinib-resistant primary CML progenitors

**Figure 5:**
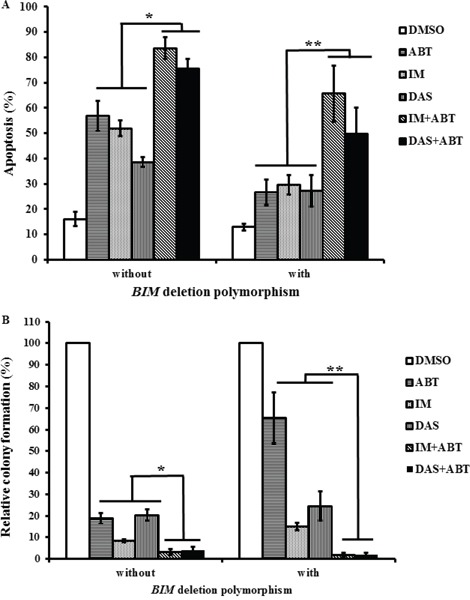
The combination of a TKI and ABT-737 induced maximal apoptosis and reduce the progenitor population in imatinib-resistant primary CML cells Cells were treated with different TKIs, alone or in combination with ABT-737 (ABT, 100 nM), for 96 hours. The TKIs used were imatinib (IM, 5 uM) and dasatinib (DAS, 100 nM). Primary CML cells with or without the *BIM* deletion polymorphism are indicated in the figure. **A.** The percentage of apoptotic cells was assessed by flow cytometry and AnnexinV/PI staining. Results are given as mean +/− s.e.m (*n* = 3). For a given sample, the calculation of the statistical significance of all combination treatments when compared to all single agent treatments was based on Student's *t* test. *P = 0.000002, **P = 0.0019. **B.** Colony-forming assay for the different primary CML cells. Relative colony formation was calculated as a percentage of the number of colonies observed in each treated sample relative to that of the respective DMSO control for each primary sample. Results are given as mean +/− s.e.m (*n* = 3). For a given sample, the calculation of the statistical significance of all combination treatments when compared to all single agent treatments was based on Student's *t* test. *P = 0.00018, **P = 0.0034.

Since the combination of a TKI and ABT-737 overcame resistance in *BIM* deletion polymorphism-containing K562 clones, feasibility of this combination in imatinib-resistant primary CML cells, with or without the *BIM* deletion polymorphism, was assessed. Imatinib and dasatinib were used at concentrations (5 uM and 100 nM respectively) corresponding to the maximum plasma levels achievable in patients [20, 21, 32]. For all single agent treatment, the average net increase in apoptosis, for primary CML cells without the *BIM* deletion polymorphism, was 33% and for those with the polymorphism, it was 15% (Figure 5A). Thus, cells with the *BIM* deletion polymorphism were significantly more resistant to single agent treatment than those without (Supplementary Table S2). For combined treatment with both TKI and ABT-737, the average increase in apoptosis, relative to the average of all single agent treatment, for primary CML cells without the polymorphism, was 92% and for those with the polymorphism, the average increase in apoptosis, was 201% (Figure 5A). Thus, treatments with both TKI and ABT-737 induced significantly more apoptosis than those with single agents in all primary CML cells especially in cells with the *BIM* deletion polymorphism where the relative increase was more than twice that of cells without the polymorphism (Figure 5A).

To assess the viability of the leukemic progenitor population, colony-forming assays were performed on these primary CML cells that were treated with either or both TKIs and ABT-737. When cells were treated with ABT-737 only; the average reduction in colony formation, relative to the DMSO control, for cells with or without the polymorphism were 81% and 35% respectively (Figure 5B). When cells were treated with imatinib only; the average reduction in colony formation, relative to the DMSO control, for cells with or without the polymorphism were 92% and 85% respectively (Figure 5B). Thus, single treatment with either ABT-737 or imatinib significantly reduced the population of progenitors without the *BIM* deletion polymorphism when compared to those with the *BIM* deletion polymorphism (Supplementary Table S2). When cells without the polymorphism were treated with both TKI and ABT-737; the average viability, relative to the average of all single agents, was further reduced by 4.4-fold and for those with the polymorphism, average viability was further reduced by 18.8-fold (Figure 5B). Again, the combined treatment with TKI and ABT-737 did have more impact on reducing progenitor population in primary CML cells with the *BIM* deletion polymorphism when compared to those without.

In summary, when compared to single agent treatment, the combination treatment with TKI and ABT-737 induced maximal apoptosis in imatinib-resistant primary CML cells especially in those that harbored the *BIM* deletion polymorphism. Importantly, the colony-forming assays performed on these primary CML cells indicated that the combined treatment with TKI and ABT-737 significantly reduced the viability of the progenitor population.

## DISCUSSION

The response of a cancer to a given therapy can be determined by both germline polymorphisms and tumor-specific acquired somatic events. We previously showed that a germline deletion polymorphism in the *BIM* gene was sufficient to mediate intrinsic resistance to TKIs in both CML and EGFR-NSCLC [1]. The *BIM* deletion polymorphism resulted in the preferential generation of *BIM* splice forms that lacked the pro-apoptotic BH3 domain and were therefore unable to induce apoptosis in response to TKI therapy. In order to study in detail how germline polymorphisms and acquired somatic events could potentially interact to cause TKI resistance in CML, we generated and characterized genome-edited K562 clones, either with or without the *BIM* deletion polymorphism, that were rendered resistant to imatinib.

*BIM* deletion polymorphism-containing imatinib-resistant clones exhibited significant increased viability when compared to their non-*BIM* deletion polymorphism-containing counterparts (Figure 2A). This increased viability was not due to changes in the proliferation rate (Figure 2B) but was due to impaired apoptosis in the *BIM* deletion polymorphism-containing imatinib-resistant cells (Figures 2C and 2D). Additionally, the *BIM* deletion polymorphism was found to be permissive for the acquisition of somatic events that mediate both BCR-ABL1-dependent and -independent mechanisms of TKI resistance (Figure 3 and Table 2). Furthermore, these acquired somatic events by the *BIM* deletion polymorphism-containing imatinib-resistant clones were distinct from those without the *BIM* deletion polymorphism (Figure 3 and Table 2). The *BIM* deletion polymorphism-containing imatinib-resistant clones incorporated activated LYN kinase and TKI-resistance associated BCR-ABL1 somatic mutations as additional TKI resistance mechanisms (Figure 3 and Table 2). Interestingly, BCR-ABL1 kinase domain mutations that were associated with TKI-resistance were also observed in 15% of CML patients with the *BIM* deletion polymorphism who experienced sub-optimal TKI responses [1].

Since the *BIM* deletion polymorphism cooperated with both BCR-ABL1-dependent and -independent mechanisms of TKI resistance, we assessed whether second-generation TKIs, that could overcome activated LYN and TKI-resistance associated BCR-ABL1 somatic mutations, could be used as a therapeutic strategy to overcome these acquired TKI resistance mechanisms. Furthermore, we also assessed the use of the BH3-mimetic ABT-737 in these imatinib-resistant clones as we previously showed that ABT-737 and imatinib resensitized parental K562 with the *BIM* deletion polymorphism to imatinib-induced apoptosis [1]. Additionally, we found that ABT-737 significantly enhanced imatinib-induced reduction in viability of not only the parental clones, but also their corresponding imatinib-resistant K562 clones (supplementary Figures S1 and S2). Thus, we went on to determine whether treatment with second-generation TKIs and/or ABT-737 could overcome TKI resistance in *BIM* deletion polymorphism-containing imatinib-resistant clones.

We found that the use of second-generation TKIs and BH3 mimetic ABT-737 had a significant effect on overcoming TKI resistance in imatinib-resistant clones, especially in those that harbored the *BIM* deletion polymorphism (Figure 4 and Supplementary Table S1). Thus, the combination of a TKI with ABT-737 represents a potential therapeutic strategy to overcome TKI resistance in CML cell lines, especially those that harbored the *BIM* deletion polymorphism. Indeed, as with the imatinib-resistant cell clones, we found that the combination treatment with TKI and ABT-737 induced maximal apoptosis in imatinib-resistant primary CML cells, especially in those with the *BIM* deletion polymorphism (Figure 5A). More importantly, based on the colony-forming assays, combination treatment with TKI and ABT-737 significantly reduced the viability of the progenitor population of these imatinib-resistant primary CML cells (Figure 5B).

In conclusion, we show that germline and acquired genetic factors can interact to produce high levels of resistance to cancer targeted therapies, and that the cell line-based approach we employed was useful to both elucidate the mechanisms underlying resistance, as well as devise therapeutic strategies to overcome such resistance. The data presented here suggest that the combined use of TKI with BH3 mimetics, such as ABT-737, could potentially be an alternative therapeutic strategy to overcome TKI-resistance in CML patients.

## MATERIALS AND METHODS

### Generation of imatinib-resistant genome-edited K562 clones

The generation of imatinib-resistant K562 clones was based on the protocol by Mahon et al; 2000 [14]. K562 clones, either with or without the genome-edited *BIM* deletion polymorphism, were initially exposed to 100 nM of imatinib. They were then grown in increasing concentrations of imatinib at a rate of 0.1 μM increment every 7–10 days of culture. Once imatinib-resistant K562 clones became resistant to 1 μM of IM, the rate of exposure was increased to 0.2–0.4 μM increments every 7–10 days of culture until they became resistant to 3 μM of IM. These imatinib-resistant K562 clones were then cultured, for long-term, at 3 μM of IM. As controls, IM-sensitive parental clones were cultured in parallel without IM. Cells were cultured in RPMI-1640 medium supplemented with penicillin/streptomycin, glutamine, and 20% FBS. Cells were kept in a humidified incubator at 37°C with 5% CO_2_.

### Chemical reagents

Drugs used were imatinib, nilotinib (both from Novartis, Switzerland), dasatinib (Bristol-Myers Squibb, UK) and ABT-737 (Chemietek, USA). The drugs were dissolved in DMSO (50% for imatinib; 100% for dasatinib, nilotinib, and ABT-737), and kept at − 20°C in aliquots.

### Fluorescence *in situ* hybridisation (FISH)

To detect *BCR-ABL1,* we used the method previously described [1].

### Cell viability assay

As previously described [33], cell viability assay was performed by using MTS tetrazolium (Cell Titer96 Aqueous; Promega, Madison, WI, USA).

### Cell cycle and apoptosis analyses by flow cytometry

Protocols for cell cycle analysis of imatinib-resistant K562 clones [33] and apoptosis assay for primary CML samples have been previously described [1].

### ELISA-based DNA fragmentation assay

The presence of mono- and oligo-nucleosomes in the apoptotic cells was detected using the Cell Death Detection ELISA (Roche, Switzerland), following the manufacturer's instructions, and as described elsewhere [1].

### Immunoblotting

We used the following antibodies for immunoblotting: BCR-ABL1 (#2802), pBCR-ABL1 (#2861), BIM (#2819), cleaved CASPASE 3 (#9661), STAT5A (#9310), pSTAT5A (#9359), PARP (#9542), pERK (#4377), ERK (#9102), MCL-1 (#4572) (all from Cell Signaling Technology), Lyn (G-7, Santa Cruz, USA), pLYN (Epitomics, USA), BCL-2 (AbCam, UK) and β-actin (#AC-15, Sigma, USA). The antibody dilutions used were 1 in 1,000; except for β-actin (1 in 5,000). HRP-conjugated secondary antibodies were specific to rabbit (Sigma) or mouse IgG (Santa Cruz biotechnology). The protein bands on the membrane were visualized using the Western Lightning chemiluminescence reagent (PerkinElmer, USA).

### Drug combination studies and BLISS fractional independence analysis

1:4 serially diluted imatinib and ABT-737 were added in combination to 96-well assay plates and media only was used as a control. Cells were seeded into the assay plates, treated and incubated at 37°C 10% CO2 for 48 hours. Cell Titer-Glo assay (Promega, USA) was used as acell viability read out. Luminescence was measured according to the manufacturer's protocol and analysed using Envision 2103 multi-label plate reader (Perkin Elmer, USA). Luminescence reading for each sample was normalized to that of untreated sample. Bliss fractional independence analysis was used to calculate the predictive additive drug responses according to this formula: Ft = Fa + Fb (1 − Fa) = Fa + Fb – (Fa * Fb), where Ft = total predicted fractional response; Fa = fraction of cells responding to drug A; Fb = fraction of cells responding to drug B; (1 − Fa) = fraction of cells that do not respond to drug A. Bliss Independence Score was calculated according to the difference between observed and predicted additive responses: Score < 0: antagonistic; Score = 0: additive; Score > 0: synergistic.

### Ethics committee approval

Clinical CML samples were obtained from the Singapore General Hospital. Written informed consent and institutional review board approval were obtained from the relevant individuals and institutions.

### Primary CML peripheral blood mononuclear cells (PBMCs) & colony formation assay

PBMCs from a total of six chronic-phase CML patients (three of them do not have the *BIM* deletion polymorphism while the others do) were used. The presence of the *BIM* deletion polymorphism was detected using the method described previously [1]. PBMCs were thawed and allowed to recover overnight in serum-free StemPro media (Invitrogen, USA), supplemented with human growth factors [15], and 1X nutrient supplement (Invitrogen, USA) [34]. Cells were then subjected to drug treatment for 96 hours in the liquid media, harvested, washed, and seeded in methylcellulose (H4434; Stemcell Technologies, USA). The aim of this assay is to determine how the viability of the primary CML cells was affected during the 96-hour incubation with various drugs in the liquid media. Colonies were enumerated after 14 days.

## SUPPLEMENTARY FIGURES AND TABLES


