# Supplementary material for: The BIM deletion polymorphism: A paradigm of a permissive interaction between germline and acquired TKI resistance factors in chronic myeloid leukemia
Source: Oncotarget. 2015 Oct 28;7(3):2721–33. doi: 10.18632/oncotarget.5436 (PMC4823067; doi:10.18632/oncotarget.5436)
Supplement: Supplementary file 1 [file oncotarget-07-2721-s001.pdf]

SUPPLEMENTARY FIGURES AND TABLES

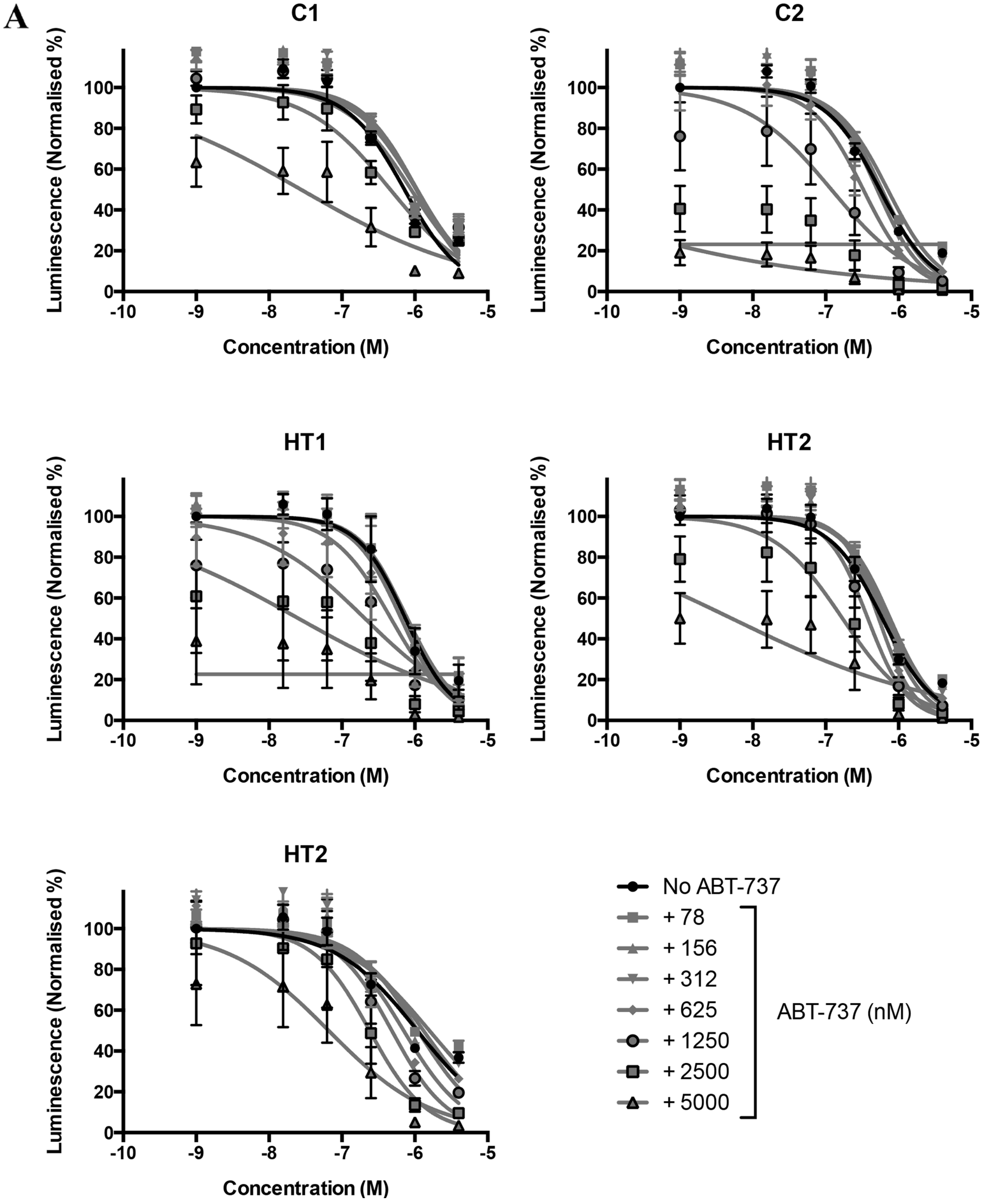

Supplementary Figure S1: The effect of different combinations of concentrations of imatinib and ABT-737 on the viability of parental (panel A) and corresponding imatinib-resistant clones (panel B). Luminescence readings are readouts for cell vability and were derived from the Cell Titer-Glo® assay (see Method section for details). The luminescence reading for each sample was normalized to that of the respective untreated sample. Results are given as mean +/− s.e.m (n = 3). The different concentrations of ABT-737 and imatinib used are indicated in the figure. (*Continued*)

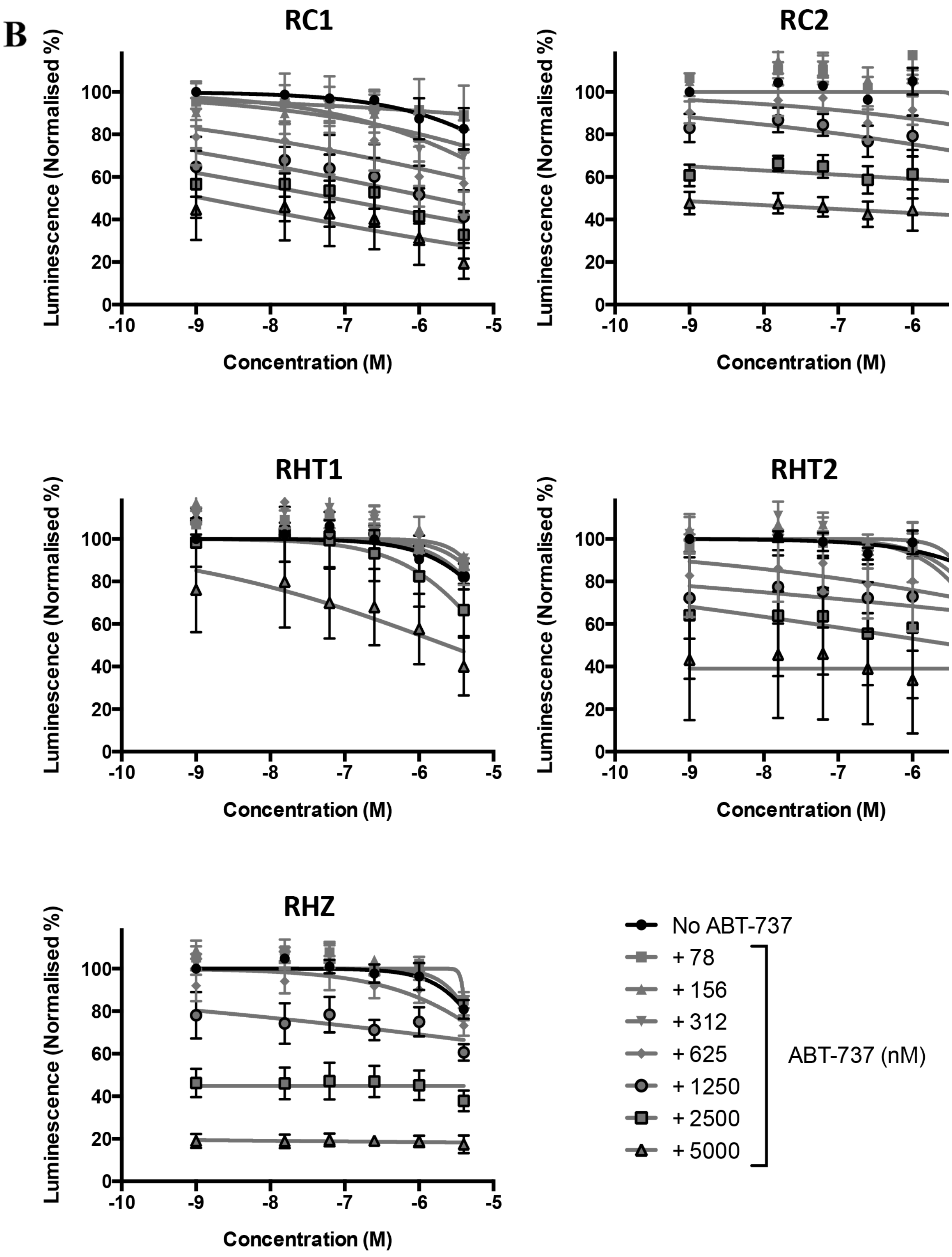

Supplementary Figure S1: (*Continued*) The effect of different combinations of concentrations of imatinib and ABT-737 on the viability of parental (panel A) and corresponding imatinib-resistant clones (panel B). Luminescence readings are readouts for cell vability and were derived from the Cell Titer-Glo® assay (see Method section for details). The luminescence reading for each sample was normalized to that of the respective untreated sample. Results are given as mean +/− s.e.m (n = 3). The different concentrations of ABT-737 and imatinib used are indicated in the figure.

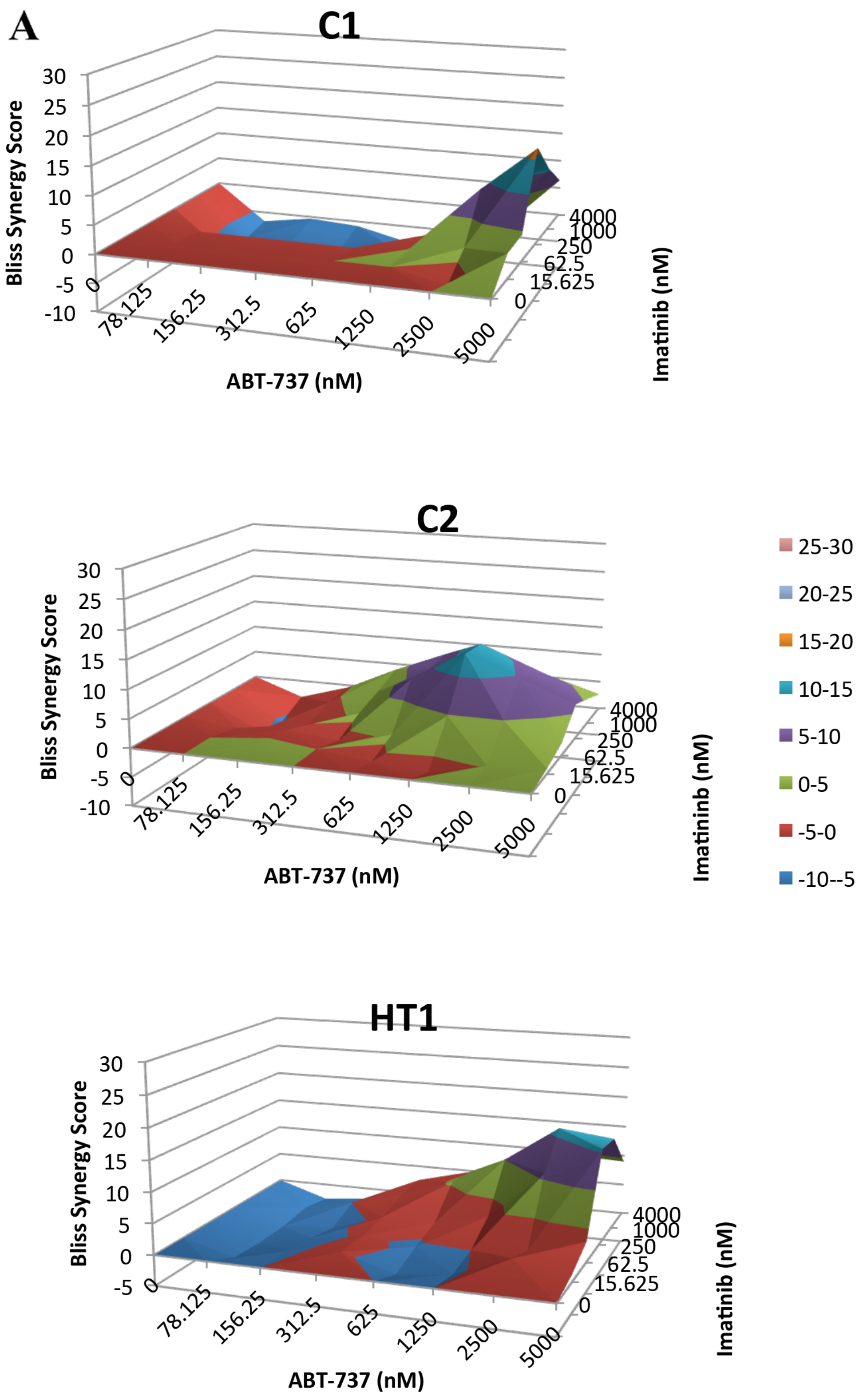

Supplementary Figure S2: Bliss fractional independence analyses on the combinatorial effect of imatinib and ABT-737 on the viability of parental (panel A) and corresponding imatinib-resistant clones (panel B). Bliss fractional independence analysis was used to calculate the predictive additive drug responses (see Methods). The Bliss Independence Score was calculated according to the difference between observed and predicted additive responses: Score < 0 : antagonistic; Score = 0 : additive; Score > 0 : synergistic. (*Continued*)

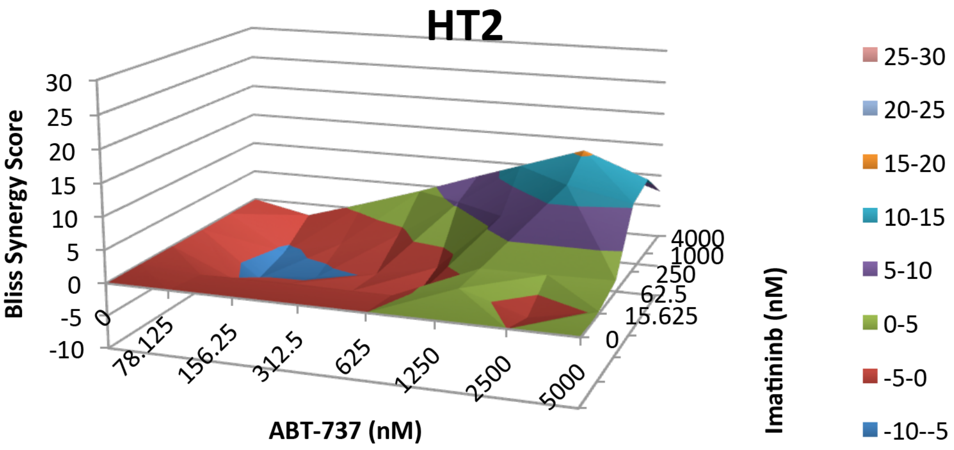

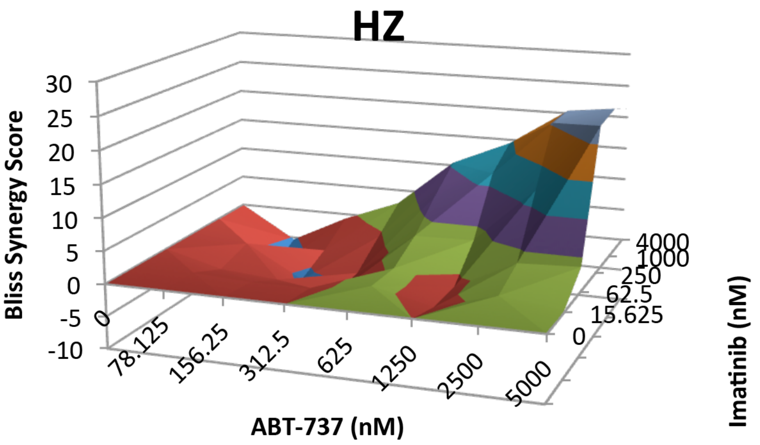

Supplementary Figure S2: (Continued) Bliss fractional independence analyses on the combinatorial effect of imatinib and ABT-737 on the viability of parental (panel A) and corresponding imatinib-resistant clones (panel B). Bliss fractional independence analysis was used to calculate the predictive additive drug responses (see Methods). The Bliss Independence Score was calculated according to the difference between observed and predicted additive responses: Score < 0 : antagonistic; Score = 0 : additive; Score > 0 : synergistic. (*Continued*)

B

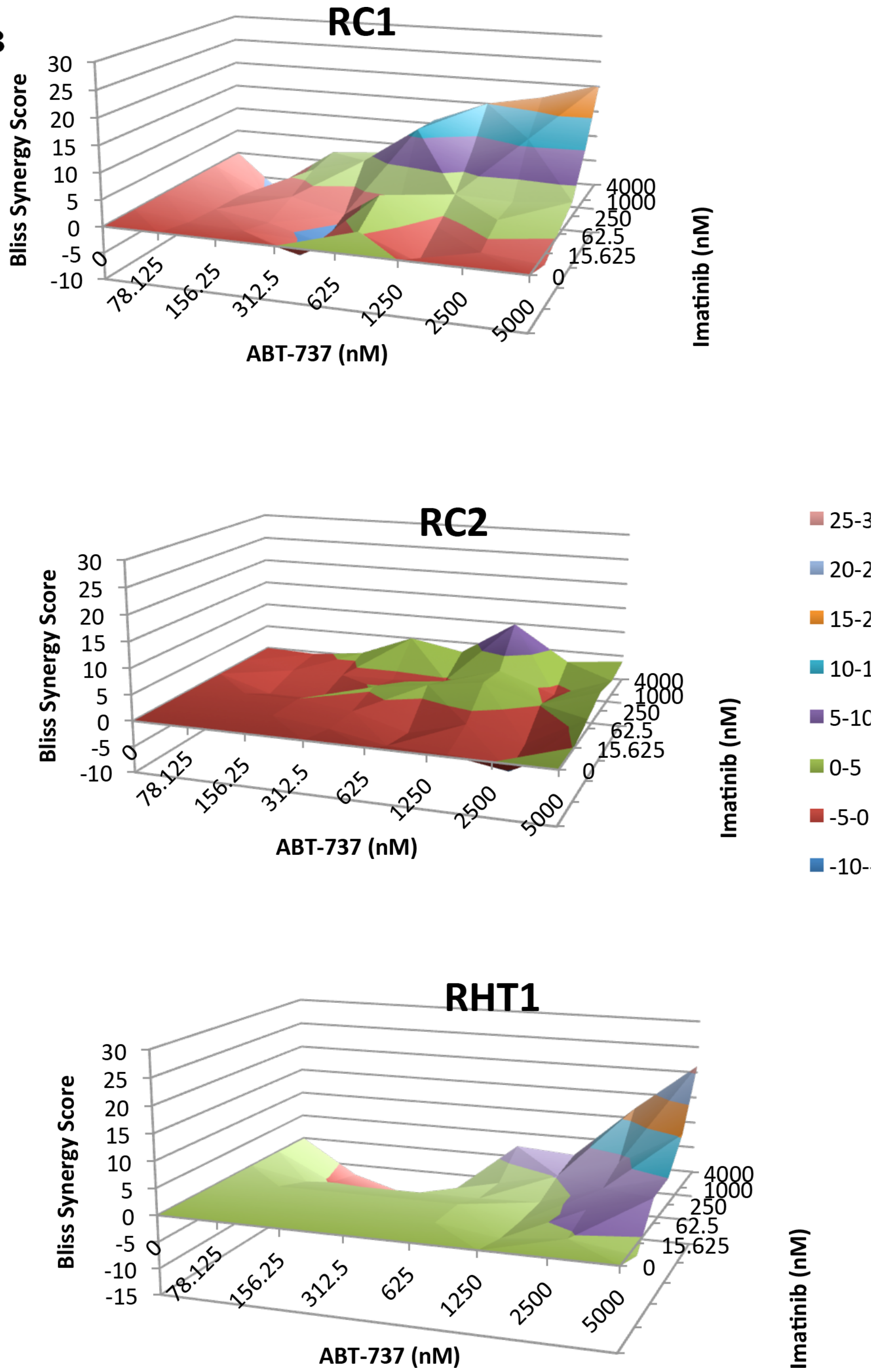

Supplementary Figure S2: (*Continued*) Bliss fractional independence analyses on the combinatorial effect of imatinib and ABT-737 on the viability of parental (panel A) and corresponding imatinib-resistant clones (panel B). Bliss fractional independence analysis was used to calculate the predictive additive drug responses (see Methods). The Bliss Independence Score was calculated according to the difference between observed and predicted additive responses: Score < 0 : antagonistic; Score = 0 : additive; Score > 0 : synergistic. (*Continued*)

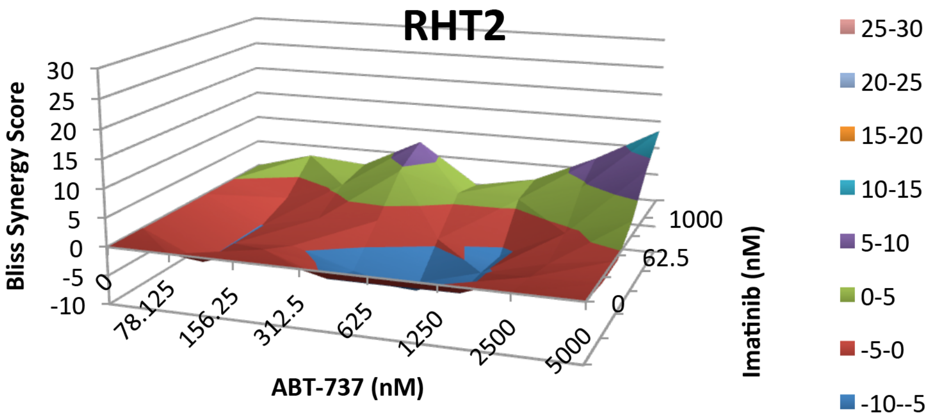

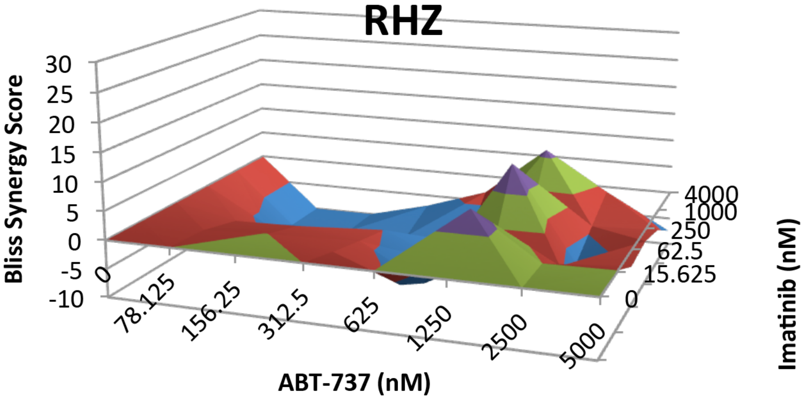

Supplementary Figure S2: (*Continued*) Bliss fractional independence analyses on the combinatorial effect of imatinib and ABT-737 on the viability of parental (panel A) and corresponding imatinib-resistant clones (panel B). Bliss fractional independence analysis was used to calculate the predictive additive drug responses (see Methods). The Bliss Independence Score was calculated according to the difference between observed and predicted additive responses: Score < 0 : antagonistic; Score = 0 : additive; Score > 0 : synergistic.

Supplementary Table S1: The use of both second-generation tyrosine kinase inhibitors, dasatinib (DAS) and nilotinib (NIL); and BH3 mimetic ABT-737 (ABT) significantly enhanced apoptosis in imatinib-resistant (IMR) cells when compared to imatinib (IM) alone (see Figure 4A)

	***P* value of different treatment regimen**				
IMR cells	DAS	NIL	IM+ABT	DAS+ABT	NIL+ABT
RC1	0.0029	ns	ns	0.013	0.033
RC2	ns	0.038	0.019	0.022	0.014
RHT1	0.0055	0.01	0.027	0.0019	0.0077
RHT2	0.014	0.001	0.0034	0.0095	0.0068
RHZ	0.00033	0.00088	0.00019	0.00001	0.013

The *P* values, calculated from Student's *t* test, for each of the treatment regimen when compared to imatinib alone are shown below. ns = not significant ($P > 0.05$)

Supplementary Table S2: Single treatment with either ABT-737 or imatinib significantly reduced the population of primary CML cells without the *BIM* deletion polymorphism when compared to those with the polymorphism

	***P* value of different treatment regimen (without polymorphism vs with polymorphism)**						
	ABT	IM	DAS	All single	ABT + IM	ABT + DAS	All combination
FACS	0.0047	0.006	ns	0.0079	ns	ns	0.015
CFA	0.018	0.011	ns	0.047	ns	ns	0.022

Student's *t* tests were performed to determine the statistical significance of each treatment regimen used on primary CML cells without the *BIM* deletion polymorphism when compared to those with the *BIM* deletion polymorphism (see figures 5A & 5B). ns= not significant ($P > 0.05$)

Supplementary Table S3: FISH analysis for the presence of BCR-ABL1 gene amplification

**Cells**	**No of cells with BCR-ABL1 gene amplification**	
	**No**	**Yes**
HZ	9	9
RHZ	4	21

RHZ cells had significantly more BCR-ABL1 gene amplification than HZ cells ($P = 0.017$). Chi-square test was used to calculate the *P*-value
